# Supplementary material for: Persistent heat waves projected for Middle East and North Africa by the end of the 21st century
Source: PLoS One. 2020 Nov 17;15(11):e0242477. doi: 10.1371/journal.pone.0242477 (PMC7671526; doi:10.1371/journal.pone.0242477)
Supplement: S1 Table — (DOCX) [file pone.0242477.s001.docx]

| **Model** | **Institute** | **RCM** | **Driving GCM** |
| --- | --- | --- | --- |
|  | CLMcom | CCLM4-8-17 | CNRM-CERFACS-CNRM-CM5 |
|  | CLMcom | CCLM4-8-17 | MOHC-HadGEM2-ES |
|  | CLMcom | CCLM4-8-17 | MPI-M-MPI-ESM-LR |
|  | KNMI | RACMO22T | ICHEC-EC-EARTH |
|  | MPI-CSC | REMO2009 | MPI-M-MPI-ESM-LR |
|  | SMHI | RCA4 | CNRM-CERFACS-CNRM-CM5 |
|  | SMHI | RCA4 | CSIRO-QCCCE-CSIRO-Mk3-6-0 |
|  | SMHI | RCA4 | IPSL-IPSL-CM5A-MR |
|  | SMHI | RCA4 | MIROC-MIROC5 |
|  | SMHI | RCA4 | MOHC-HadGEM2-ES |
|  | SMHI | RCA4 | MPI-M-MPI-ESM-LR |
|  | SMHI | RCA4 | NCC-NorESM1-M |
|  | SMHI | RCA4 | NOAA-GFDL-GFDL-ESM2M |

**Table S1.** List of models from CORDEX
