# Supplementary material for: Persistent heat waves projected for Middle East and North Africa by the end of the 21st century
Source: PLoS One. 2020 Nov 17;15(11):e0242477. doi: 10.1371/journal.pone.0242477 (PMC7671526; doi:10.1371/journal.pone.0242477)
Supplement: S2 Table — RCP4.5 and RCP8.5 refer to the two representative concentration pathways used during the calculations. All the results are statistically significant at more than 95%. (DOCX) [file pone.0242477.s002.docx]

| **CITY** | **MONTH** | **MAXIMUM TEMPERATURE** | | | | |
| --- | --- | --- | --- | --- | --- | --- |
|  |  | **LATE 20^TH^ CENTURY**  **(1970-1999)** | **RCP4.5**  **(2020-2049)** | **RCP4.5**  **(2070-2099)** | **RCP8.5**  **(2020-2049)** | **RCP8.5**  **(2070-2099)** |
| 1. Abidjan (Ivory Coast) | 2,3,4 | 33.8 ± 1.5 | 34.2 ± 1.9 | 35.3 ± 1.9 | 34.4 ± 2.0 | 37.0 ± 2.0 |
| 1. Abu Dhabi (Emirates) | 6,7,8 | 39.0 ± 1.9 | 40.6 ± 2.0 | 41.6 ± 2.1 | 40.8 ± 2.0 | 43.7 ± 2.2 |
| 1. Abuja (Nigeria) | 2,3,4 | 34.6 ± 1.8 | 36.1 ± 2.0 | 37.2 ± 2.1 | 36.3 ± 2.0 | 39.1 ± 2.3 |
| 1. Accra (Ghana) | 2,3,4 | 31.7 ± 1.5 | 32.9 ± 1.6 | 33.8 ± 1.6 | 33.0 ± 1.6 | 35.4 ± 1.7 |
| 1. Addis Ababa (Ethiopia) | 3,4,5 | 22.9 ± 1.7 | 24.6 ± 1.7 | 25.9 ± 1.9 | 25.0 ± 1.8 | 28.1 ± 2.0 |
| 1. Alexandria (Egypt) | 6,7,8 | 30.4 ± 1.5 | 31.9 ± 1.7 | 32.4 ± 1.9 | 32.2 ± 1.7 | 33.9 ± 1.9 |
| 1. Algiers (Algeria) | 7,8,9 | 29.0 ± 1.4 | 30.7 ± 1.6 | 31.9 ± 1.8 | 30.9 ± 1.6 | 34.0 ± 2.0 |
| 1. Amman (Jordan) | 7,8,9 | 34.6 ± 1.0 | 36.3 ± 1.1 | 37.4 ± 1.1 | 36.6 ± 1.1 | 39.7 ± 1.3 |
| 1. Ankara (Turkey) | 6,7,8 | 26.3 ± 1.5 | 28.5 ± 1.6 | 29.9 ± 1.8 | 28.9 ± 1.7 | 32.9 ± 1.8 |
| 1. Ashgabat (Turkmenistan) | 6,7,8 | 34.6 ± 1.1 | 36.5 ± 1.2 | 37.9 ± 1.3 | 36.8 ± 1.2 | 40.5 ± 1.5 |
| 1. Asmara (Eritrea) | 4,5,6 | 28.9 ± 1.6 | 30.7 ± 1.7 | 32.0 ± 1.8 | 31.0 ± 1.7 | 34.2 ± 2.0 |
| 1. Baghdad (Iraq) | 6,7,8 | 43.8 ± 1.1 | 45.8 ± 1.3 | 47.1 ± 1.4 | 46.1 ± 1.3 | 49.8 ± 1.6 |
| 1. Baku (Azerbaijan) | 6,7,8 | 30.2 ± 2.0 | 31.8 ± 2.2 | 32.9 ± 2.3 | 32.1 ± 2.2 | 35.0 ± 2.4 |
| **CITY** | **MONTH** | **MAXIMUM TEMPERATURE** | | | | |
|  |  | **LATE 20^TH^ CENTURY**  **(1970-1999)** | **RCP4.5**  **(2020-2049)** | **RCP4.5**  **(2070-2099)** | **RCP8.5**  **(2020-2049)** | **RCP8.5**  **(2070-2099)** |
| 1. Bamako (Mali) | 3,4,5 | 36.9 ± 1.9 | 38.3 ± 2.2 | 39.5 ± 2.4 | 38.5 ± 2.3 | 41.6 ± 2.6 |
| 1. Bangui (Central African Republic) | 2,3,4 | 34.7 ± 2.4 | 36.2 ± 2.6 | 37.3 ± 2.7 | 36.5 ± 2.6 | 39.3 ± 2.8 |
| 1. Banjul (The Gambia) | 2,3,11 | 34.0 ± 1.0 | 35.4 ± 1.1 | 36.2 ± 1.1 | 35.5 ± 1.1 | 38.0 ± 1.3 |
| 1. Beirut (Lebanon) | 7,8,9 | 27.6 ± 0.9 | 29.0 ± 1.0 | 30.0 ± 1.0 | 29.3 ± 1.0 | 31.9 ± 1.1 |
| 1. Bissau (Guinea-Bissau) | 3,4,5 | 34.6 ± 1.7 | 35.8 ± 1.7 | 36.6 ± 1.9 | 35.9 ± 1.8 | 38.2 ± 1.9 |
| 1. Cairo (Egypt) | 6,7,8 | 37.0 ± 0.8 | 38.7 ± 1.1 | 39.8 ± 1.2 | 39.0 ± 1.1 | 42.1 ± 1.4 |
| 1. Conakry (Guinea) | 2,3,4,5 | 28.8 ± 1.2 | 29.8 ± 1.3 | 30.6 ± 1.3 | 29.9 ± 1.3 | 32.0 ± 1.4 |
| 1. Dakar (Senegal) | 9,10,11 | 28.8 ± 1.7 | 29.9 ± 1.7 | 30.7 ± 1.7 | 30.1 ± 1.7 | 32.1 ± 1.8 |
| 1. Damascus (Syria) | 6,7,8 | 30.7 ± 1.5 | 32.6 ± 1.6 | 33.8 ± 1.7 | 32.9 ± 1.7 | 36.2 ± 1.9 |
| 1. Djibouti (Djibouti) | 6,7,8 | 36.1 ± 2.7 | 37.3 ± 2.7 | 38.3 ± 2.7 | 37.5 ± 2.7 | 40.1 ± 2.8 |
| 1. Doha (Qatar) | 6,7,8 | 37.2 ± 2.2 | 38.8 ± 2.2 | 39.8 ± 2.3 | 39.0 ± 2.3 | 41.8 ± 2.6 |
| 1. El-Aiun (Western Sahara) | 7,8,9 | 28.7 ± 0.9 | 29.8 ± 0.9 | 30.6 ± 0.8 | 29.9 ± 0.9 | 31.9 ± 0.9 |
| 1. Freetown (Sierra Leone) | 3,4,5 | 29.3 ± 1.1 | 30.4 ± 1.2 | 31.3 ± 1.3 | 30.6 ± 1.2 | 32.8 ± 1.4 |
| **CITY** | **MONTH** | **MAXIMUM TEMPERATURE** | | | | |
|  |  | **LATE 20^TH^ CENTURY**  **(1970-1999)** | **RCP4.5**  **(2020-2049)** | **RCP4.5**  **(2070-2099)** | **RCP8.5**  **(2020-2049)** | **RCP8.5**  **(2070-2099)** |
| 1. Giza (Egypt) | 6,7,8 | 36.8 ± 0.8 | 38.5 ± 1.1 | 38.9 ± 1.3 | 38.8 ± 1.1 | 41.1 ± 1.4 |
| 1. Istanbul (Turkey) | 6,7,8 | 26.7 ± 2.9 | 28.5 ± 2.9 | 28.8 ± 3.0 | 28.6 ± 3.0 | 30.5 ± 3.1 |
| 1. Jerusalem (Israel) | 7,8,9 | 34.2 ± 1.1 | 35.8 ± 1.2 | 36.9 ± 1.2 | 36.2 ± 1.2 | 39.1 ± 1.3 |
| 1. Juba (South Sudan) | 1,2,3 | 36.1 ± 2.0 | 37.6 ± 2.1 | 38.6 ± 2.3 | 37.8 ± 2.2 | 40.6 ± 2.3 |
| 1. Khartoum (Sudan) | 4,5,6 | 38.4 ± 1.7 | 40.0 ± 1.9 | 41.4 ± 2.0 | 40.3 ± 1.9 | 43.6 ± 2.1 |
| 1. Kuwait City (Kuwait) | 6,7,8 | 40.6 ± 2.0 | 42.3 ± 2.2 | 43.4 ± 2.3 | 42.5 ± 2.2 | 45.5 ± 2.4 |
| 1. Lagos (Nigeria) | 1,2,3,4 | 31.2 ± 1.2 | 32.3 ± 1.2 | 33.2 ± 1.3 | 32.4 ± 1.2 | 34.7 ± 1.4 |
| 1. Lome (Togo) | 2,3,4 | 32.0 ± 1.4 | 33.2 ± 1.5 | 34.0 ± 1.6 | 33.3 ± 1.5 | 35.6 ± 1.6 |
| 1. Manama (Bahrain) | 6,7,8,9 | 38.4 ± 2.6 | 40.1 ± 2.7 | 41.2 ± 2.9 | 40.3 ± 2.8 | 43.3 ± 3.1 |
| 1. Mogadishu (Somalia) | 3,4,5 | 29.7 ± 1.3 | 30.8 ± 1.4 | 31.6 ± 1.4 | 31.0 ± 1.4 | 33.1 ± 1.5 |
| 1. Monrovia (Liberia) | 2,3,4 | 29.3 ± 1.3 | 30.5 ± 1.4 | 31.3 ± 1.4 | 30.6 ± 1.4 | 32.9 ± 1.5 |
| 1. Muscat (Oman) | 5,6,7 | 34.5 ± 1.6 | 35.9 ± 1.7 | 37.0 ± 1.9 | 36.2 ± 1.7 | 38.9 ± 2.0 |
| 1. N’Djamena (Chad) | 3,4,5 | 38.3 ± 2.3 | 39.9 ± 2.4 | 41.2 ± 2.6 | 40.3 ± 2.5 | 43.2 ± 2.8 |
| **CITY** | **MONTH** | **MAXIMUM TEMPERATURE** | | | | |
|  |  | **LATE 20^TH^ CENTURY**  **(1970-1999)** | **RCP4.5**  **(2020-2049)** | **RCP4.5**  **(2070-2099)** | **RCP8.5**  **(2020-2049)** | **RCP8.5**  **(2070-2099)** |
| 1. Niamey (Niger) | 3,4,5 | 38.0 ± 1.9 | 39.5 ± 2.1 | 40.8 ± 2.3 | 39.8 ± 2.2 | 42.8 ± 2.5 |
| 1. Nouakchott (Mauritania) | 6,9,10 | 31.8 ± 1.2 | 32.9 ± 1.1 | 33.7 ± 1.2 | 33.0 ± 1.2 | 35.0 ± 1.3 |
| 1. Ouagadougou (Burkina Faso) | 3,4,5 | 37.3 ± 2.0 | 38.8 ± 2.2 | 40.0 ± 2.5 | 39.1 ± 2.4 | 42.0 ± 2.7 |
| 1. Porto-Novo (Benin) | 2,3,4 | 32.6 ± 1.7 | 33.8 ± 1.8 | 34.7 ± 1.9 | 33.9 ± 1.8 | 36.3 ± 2.0 |
| 1. Rabat (Morocco) | 7,8,9 | 29.8 ± 1.1 | 31.2 ± 1.3 | 32.1 ± 1.5 | 31.3 ± 1.3 | 33.7 ± 1.5 |
| 1. Riyadh (Saudi Arabia) | 6,7,8 | 41.4 ± 1.1 | 43.4 ± 1.3 | 44.7 ± 1.5 | 43.7 ± 1.2 | 47.3 ± 1.6 |
| 1. Sanaa (Yemen) | 6,7,8 | 24.4 ± 1.9 | 26.1 ± 2.1 | 27.4 ± 2.1 | 26.4 ± 2.1 | 29.9 ± 2.3 |
| 1. Tehran (Iran) | 6,7,8 | 26.4 ± 2.1 | 28.7 ± 1.9 | 30.4 ± 2.0 | 29.1 ± 1.9 | 33.2 ± 2.1 |
| 1. Tbilisi (Georgia) | 6,7,8 | 27.1 ± 2.8 | 29.2 ± 3.1 | 30.7 ± 3.1 | 29.6 ± 3.1 | 33.6 ± 3.1 |
| 1. Tripoli (Libya) | 7,8,9 | 31.5 ± 1.6 | 32.9 ± 1.6 | 33.9 ± 1.8 | 33.2 ± 1.7 | 35.9 ± 2.0 |
| 1. Tunis (Tunisia) | 7,8,9 | 31.4 ± 1.4 | 33.1 ± 1.6 | 34.3 ± 1.8 | 33.3 ± 1.6 | 36.5 ± 2.1 |
| 1. Yamoussoukro (Ivory Coast) | 2,3,4 | 34.8 ± 2.1 | 36.1 ± 2.2 | 37.1 ± 2.3 | 36.3 ± 2.3 | 39.0 ± 2.5 |
| 1. Yaounde (Cameroon) | 1,2,3,4 | 29.9 ± 3.2 | 31.2 ± 3.3 | 32.2 ± 3.5 | 31.4 ± 3.4 | 34.0 ± 3.6 |
| **CITY** | **MONTH** | **MAXIMUM TEMPERATURE** | | | | |
|  |  | **LATE 20^TH^ CENTURY**  **(1970-1999)** | **RCP4.5**  **(2020-2049)** | **RCP4.5**  **(2070-2099)** | **RCP8.5**  **(2020-2049)** | **RCP8.5**  **(2070-2099)** |
| 1. Yerevan (Armenia) | 6,7,8 | 22.9 ± 1.7 | 25.1 ± 2.0 | 26.7 ± 2.1 | 25.5 ± 1.9 | 29.8 ± 2.3 |

**Table S2.** Maximum temperature (value ± SD) in ºC averaged over the periods 1970-1999, 2020-2049 and 2070-2099. RCP4.5 and RCP8.5 refer to the two representative concentration pathways used during the calculations. *All the results are statistically significant at more than 95%.*
