# Supplementary material for: Persistent heat waves projected for Middle East and North Africa by the end of the 21st century
Source: PLoS One. 2020 Nov 17;15(11):e0242477. doi: 10.1371/journal.pone.0242477 (PMC7671526; doi:10.1371/journal.pone.0242477)
Supplement: S3 Table — RCP4.5 and RCP8.5 refer to the two representative concentration pathways used during the calculations. All the results are statistically significant at more than 95%. (DOCX) [file pone.0242477.s003.docx]

| **CITY** | **PERCENTAGE OF DAYS UNDER EXTREME TEMPERATURE CONDITIONS** | | | | |
| --- | --- | --- | --- | --- | --- |
|  | **LATE 20^TH^ CENTURY**  **(1970-1999)** | **RCP4.5**  **(2020-2049)** | **RCP4.5**  **(2070-2099)** | **RCP8.5**  **(2020-2049)** | **RCP8.5**  **(2070-2099)** |
| 1. Abidjan | 4.8 ± 0.8 | 32.5 ± 11.2 | 59.3 ± 17.4 | 37.4 ±11.6 | 86.7 ± 9.8 |
| 1. Abu Dhabi | 4.7 ± 0.9 | 32.3 ± 9.1 | 54.7 ± 19.1 | 38.4 ± 10.6 | 85.3 ± 11.6 |
| 1. Abuja | 4.1 ± 0.6 | 25.7 ± 5.8 | 47.3 ± 13.0 | 28.5 ± 7.9 | 72.6 ± 11.3 |
| 1. Accra | 4.6 ± 1.0 | 43.0 ± 14.9 | 70.8 ± 15.9 | 47.5 ± 16.7 | 92.9 ± 5.2 |
| 1. Addis Ababa | 4.7 ± 0.9 | 38.1 ± 11.4 | 63.1 ± 16.2 | 46.1 ± 12.5 | 85.2 ± 11.3 |
| 1. Alexandria | 5.1 ± 0.4 | 16.5 ± 7.0 | 26.0 ± 12.6 | 20.4 ± 8.5 | 52.3 ± 13.2 |
| 1. Algiers | 5.1 ± 0.7 | 15.2 ± 6.6 | 27.2 ± 13.4 | 17.3 ± 7.1 | 53.2 ± 20.0 |
| 1. Amman | 4.8 ± 0.5 | 13.8 ± 3.9 | 23.8 ± 8.0 | 16.7 ± 5.1 | 51.5 ± 16.9 |
| 1. Ankara | 4.6 ± 0.6 | 19.5 ± 5.4 | 35.0 ± 11.5 | 22.7 ± 6.3 | 67.6 ± 14.0 |
| 1. Ashgabat | 4.9 ± 0.6 | 18.6 ± 6.8 | 34.5 ± 15.0 | 21.7 ± 9.1 | 62.9 ± 17.9 |
| 1. Asmara | 4.4 ± 0.8 | 32.2 ± 9.8 | 57.4 ± 14.6 | 38.6 ± 10.8 | 83.7 ± 10.5 |
| 1. Baghdad | 4.9 ± 0.8 | 22.7 ± 8.0 | 41.0 ± 13.8 | 27.3 ± 9.6 | 74.4 ± 16.3 |
| 1. Baku | 4.8 ± 0.7 | 16.9 ± 5.0 | 30.3 ± 12.4 | 19.9 ± 6.3 | 58.0 ± 16.7 |
| **CITY** | **PERCENTAGE OF DAYS UNDER EXTREME TEMPERATURE CONDITIONS** | | | | |
|  | **LATE 20^TH^ CENTURY**  **(1970-1999)** | **RCP4.5**  **(2020-2049)** | **RCP4.5**  **(2070-2099)** | **RCP8.5**  **(2020-2049)** | **RCP8.5**  **(2070-2099)** |
| 1. Bamako | 5.0 ± 1.0 | 28.4 ± 8.0 | 51.8 ± 14.4 | 33.1 ± 10.3 | 76.4 ± 13.5 |
| 1. Bangui | 4.4 ± 0.8 | 25.7 ± 6.9 | 46.6 ± 13.1 | 30.4 ± 8.4 | 72.0 ± 9.9 |
| 1. Banjul | 4.3 ± 1.0 | 22.7 ± 6.2 | 39.5 ± 9.0 | 25.1 ± 6.4 | 66.0 ± 10.5 |
| 1. Beirut | 4.7 ± 0.5 | 15.6 ± 5.0 | 29.0 ± 12.4 | 19.3 ± 7.0 | 62.4 ± 20.9 |
| 1. Bissau | 5.0 ± 0.5 | 18.8 ± 4.6 | 35.3 ± 12.7 | 21.4 ± 6.7 | 68.4 ± 15.4 |
| 1. Cairo | 5.0 ± 0.5 | 17.8 ± 7.1 | 33.3 ± 15.5 | 22.0 ± 9.0 | 66.1 ± 15.6 |
| 1. Conakry | 4.5 ± 0.6 | 30.7 ± 12.6 | 60.2 ± 21.3 | 35.6 ± 14.6 | 90.7 ± 11.0 |
| 1. Dakar | 4.7 ± 1.0 | 27.3 ± 10.9 | 49.6 ± 16.1 | 32.1 ± 12.1 | 77.0 ± 11.3 |
| 1. Damascus | 4.9 ± 0.7 | 16.3 ± 4.3 | 28.7 ± 8.0 | 20.0 ± 5.6 | 59.0 ± 15.5 |
| 1. Djibouti | 4.4 ± 0.9 | 26.4 ± 6.9 | 49.7 ± 12.7 | 31.2 ± 7.8 | 75.5 ± 13.8 |
| 1. Doha | 4.4 ± 0.8 | 29.2 ± 7.8 | 55.0 ± 15.0 | 34.5 ± 10.0 | 88.2 ± 10.3 |
| 1. El-Aiun | 5.2 ± 0.5 | 8.5 ± 1.5 | 11.3 ± 2.8 | 8.9 ± 2.0 | 18.8 ± 6.9 |
| 1. Freetown | 4.6 ± 0.9 | 38.2 ± 17.1 | 69.3 ± 17.3 | 43.3 ± 16.7 | 95.7 ± 9.0 |
| **CITY** | **PERCENTAGE OF DAYS UNDER EXTREME TEMPERATURE CONDITIONS** | | | | |
|  | **LATE 20^TH^ CENTURY**  **(1970-1999)** | **RCP4.5**  **(2020-2049)** | **RCP4.5**  **(2070-2099)** | **RCP8.5**  **(2020-2049)** | **RCP8.5**  **(2070-2099)** |
| 1. Giza | 5.2 ± 0.4 | 17.4 ± 6.6 | 28.5 ± 13.6 | 21.1 ± 8.0 | 54.5 ± 13.7 |
| 1. Istanbul | 4.5 ± 0.8 | 17.3 ± 8.2 | 21.6 ± 11.2 | 19.9 ± 9.3 | 44.5 ± 17.9 |
| 1. Jerusalem | 4.9 ± 0.5 | 12.6 ± 4.3 | 21.4 ± 9.7 | 15.1 ± 5.2 | 46.8 ± 19.1 |
| 1. Juba | 3.8 ± 0.7 | 31.0 ± 11.0 | 54.6 ± 17.3 | 36.3 ± 12.9 | 81.7 ± 10.9 |
| 1. Khartoum | 4.8 ± 0.8 | 27.2 ± 9.7 | 49.8 ± 13.7 | 32.8 ± 10.1 | 76.5 ± 11.6 |
| 1. Kuwait City | 4.4 ± 0.6 | 26.9 ± 6.8 | 45.6 ± 12.5 | 30.6 ± 8.6 | 74.0 ± 12.2 |
| 1. Lagos | 4.4 ± 1.0 | 42.4 ± 13.9 | 72.6 ± 19.0 | 47.1 ± 15.2 | 94.2 ± 9.0 |
| 1. Lome | 4.4 ± 0.9 | 40.6 ± 14.7 | 68.1 ± 17.2 | 44.4 ± 16.1 | 91.6 ± 6.9 |
| 1. Manama | 4.3 ± 0.4 | 25.6 ± 6.3 | 51.0 ± 11.6 | 31.9 ± 8.9 | 88.2 ± 8.1 |
| 1. Mogadishu | 4.9 ± 1.1 | 39.7 ± 9.7 | 73.9 ± 12.0 | 48.8 ± 10.5 | 97.0 ± 3.8 |
| 1. Monrovia | 4.3 ± 0.8 | 32.4 ± 10.1 | 58.5 ± 17.3 | 37.2 ± 10.9 | 87.5 ± 9.0 |
| 1. Muscat | 4.2 ± 0.5 | 25.1 ± 6.1 | 48.9 ± 12.5 | 31.3 ± 7.7 | 82.4 ± 9.7 |
| 1. N’Djamena | 4.4 ± 0.8 | 23.3 ± 5.9 | 41.5 ± 10.2 | 28.2 ± 7.8 | 66.0 ± 11.9 |
| **CITY** | **PERCENTAGE OF DAYS UNDER EXTREME TEMPERATURE CONDITIONS** | | | | |
|  | **LATE 20^TH^ CENTURY**  **(1970-1999)** | **RCP4.5**  **(2020-2049)** | **RCP4.5**  **(2070-2099)** | **RCP8.5**  **(2020-2049)** | **RCP8.5**  **(2070-2099)** |
| 1. Niamey | 4.6 ± 0.9 | 26.0 ± 7.1 | 45.9 ± 12.1 | 30.2 ± 7.9 | 70.2 ± 12.3 |
| 1. Nouakchott | 3.9 ± 0.8 | 14.7 ± 3.1 | 24.5 ± 7.2 | 16.4 ± 3.1 | 39.9 ± 10.5 |
| 1. Ouagadougou | 4.6 ± 1.0 | 25.1 ± 6.6 | 45.5 ± 13.3 | 29.1 ± 8.7 | 70.4 ± 13.6 |
| 1. Porto-Novo | 4.5 ± 0.8 | 36.1 ± 11.7 | 63.1 ± 14.4 | 40.2 ± 13.2 | 88.6 ± 7.5 |
| 1. Rabat | 4.9 ± 0.6 | 11.2 ± 2.8 | 16.4 ± 5.6 | 12.1 ± 3.5 | 25.5 ± 9.9 |
| 1. Riyadh | 4.3 ± 0.8 | 42.2 ± 12.9 | 72.2 ± 18.1 | 49.6 ± 14.1 | 94.3 ± 8.8 |
| 1. Sanaa | 4.3 ± 0.8 | 30.3 ± 12.9 | 54.7 ± 19.1 | 35.3 ± 13.2 | 80.4 ± 15.4 |
| 1. Tehran | 4.3 ± 0.9 | 26.5 ± 9.3 | 49.7 ± 17.4 | 31.7 ± 11.6 | 79.7 ± 14.2 |
| 1. Tbilisi | 4.9 ± 0.8 | 20.4 ± 6.2 | 37.5 ± 12.4 | 24.3 ± 7.1 | 68.5 ± 15.6 |
| 1. Tripoli | 5.4 ± 0.4 | 10.1 ± 2.3 | 15.5 ± 5.8 | 11.6 ± 2.5 | 31.9 ± 14.5 |
| 1. Tunis | 5.1 ± 0.5 | 12.4 ± 5.1 | 22.0 ± 12.2 | 14.2 ± 5.9 | 44.4 ± 19.0 |
| 1. Yamoussoukro | 4.5 ± 0.7 | 24.1 ± 6.9 | 44.9 ± 12.1 | 27.6 ± 7.2 | 74.4 ± 10.6 |
| 1. Yaounde | 4.3 ± 0.6 | 28.2 ± 8.2 | 51.9 ± 15.5 | 32.4 ± 10.1 | 79.9 ± 9.6 |
| **CITY** | **PERCENTAGE OF DAYS UNDER EXTREME TEMPERATURE CONDITIONS** | | | | |
|  | **LATE 20^TH^ CENTURY**  **(1970-1999)** | **RCP4.5**  **(2020-2049)** | **RCP4.5**  **(2070-2099)** | **RCP8.5**  **(2020-2049)** | **RCP8.5**  **(2070-2099)** |
| 1. Yerevan | 5.0 ± 0.8 | 21.1 ± 6.8 | 39.6 ± 12.9 | 25.5 ± 8.0 | 71.3 ± 15.8 |

**Table S3.** Percentage of days (value ± SD) under extreme temperature conditions per month and averaged over the periods 1970-1999, 2020-2049 and 2070-2099. RCP4.5 and RCP8.5 refer to the two representative concentration pathways used during the calculations. *All the results are statistically significant at more than 95%.*
