# Supplementary material for: Persistent heat waves projected for Middle East and North Africa by the end of the 21st century
Source: PLoS One. 2020 Nov 17;15(11):e0242477. doi: 10.1371/journal.pone.0242477 (PMC7671526; doi:10.1371/journal.pone.0242477)
Supplement: S4 Table — RCP4.5 and RCP8.5 refer to the two representative concentration pathways used during the calculations. All the results are statistically significant at more than 95%. (DOCX) [file pone.0242477.s004.docx]

| **CITY** | **PERCENTAGE OF DAYS UNDER HEAT WAVE CONDITIONS** | | | | |
| --- | --- | --- | --- | --- | --- |
|  | **LATE 20^TH^ CENTURY**  **(1970-1999)** | **RCP4.5**  **(2020-2049)** | **RCP4.5**  **(2070-2099)** | **RCP8.5**  **(2020-2049)** | **RCP8.5**  **(2070-2099)** |
| 1. Abidjan | 2.2 ± 0.9 | 25.0 ± 12.3 | 51.8 ± 20.9 | 29.7 ± 13.0 | 83.7 ± 12.3 |
| 1. Abu Dhabi | 1.6 ± 0.6 | 24.4 ± 10.1 | 48.4 ± 16.5 | 30.4 ± 12.1 | 83.0 ± 13.4 |
| 1. Abuja | 1.9 ± 0.4 | 20.5 ± 6.9 | 37.7 ± 12.1 | 22.9 ± 9.0 | 69.9 ± 12.3 |
| 1. Accra | 2.0 ± 0.7 | 35.0 ± 16.6 | 65.1 ± 19.4 | 40.1 ± 18.4 | 91.7 ± 6.3 |
| 1. Addis Ababa | 2.6 ± 0.9 | 33.7 ± 12.2 | 59.7 ± 17.3 | 41.6 ± 13.4 | 83.2 ± 12.8 |
| 1. Alexandria | 1.8 ± 0.5 | 11.3 ± 6.6 | 20.2 ± 13.0 | 15.0 ± 8.5 | 47.2 ± 14.5 |
| 1. Algiers | 1.7 ± 0.5 | 8.8 ± 6.0 | 19.4 ± 14.1 | 10.4 ± 6.8 | 46.6 ± 22.5 |
| 1. Amman | 2.6 ± 0.7 | 9.9 ± 3.6 | 18.8 ± 8.0 | 12.5 ± 4.9 | 46.7 ± 18.4 |
| 1. Ankara | 2.5 ± 0.7 | 14.6 ± 5.5 | 29.3 ± 12.3 | 17.4 ± 6.2 | 64.3 ± 15.3 |
| 1. Ashgabat | 2.0 ± 0.4 | 12.4 ± 6.7 | 27.7 ± 16.3 | 15.4 ± 9.1 | 58.1 ± 20.4 |
| 1. Asmara | 2.5 ± 0.6 | 26.9 ± 10.5 | 53.2 ± 16.1 | 34.0 ± 11.5 | 81.6 ± 12.0 |
| 1. Baghdad | 2.9 ± 0.9 | 18.4 ± 8.1 | 35.9 ± 14.6 | 22.5 ± 9.7 | 71.5 ± 18.0 |
| 1. Baku | 2.1 ± 0.8 | 10.9 ± 4.7 | 23.0 ± 13.1 | 13.7 ± 6.2 | 51.3 ± 18.8 |
| **CITY** | **PERCENTAGE OF DAYS UNDER HEAT WAVE CONDITIONS** | | | | |
|  | **LATE 20^TH^ CENTURY**  **(1970-1999)** | **RCP4.5**  **(2020-2049)** | **RCP4.5**  **(2070-2099)** | **RCP8.5**  **(2020-2049)** | **RCP8.5**  **(2070-2099)** |
| 1. Bamako | 2.6 ± 0.9 | 23.3 ± 7.4 | 47.5 ± 15.1 | 27.8 ± 10.1 | 73.8 ± 14.6 |
| 1. Bangui | 2.0 ± 0.6 | 18.8 ± 6.8 | 38.2 ± 13.5 | 22.7 ± 8.5 | 65.6 ± 11.3 |
| 1. Banjul | 1.7 ± 0.7 | 16.8 ± 5.9 | 33.8 ± 9.4 | 19.2 ± 6.1 | 62.5 ± 11.6 |
| 1. Beirut | 2.5 ± 0.5 | 11.7 ± 4.8 | 24.4 ± 12.5 | 15.0 ± 6.5 | 58.8 ± 22.5 |
| 1. Bissau | 1.5 ± 0.6 | 11.4 ± 4.3 | 26.9 ± 13.6 | 13.5 ± 6.3 | 62.7 ± 18.3 |
| 1. Cairo | 2.5 ± 0.5 | 13.5 ± 6.9 | 28.3 ± 16.2 | 17.4 ± 9.0 | 62.8 ± 17.2 |
| 1. Conakry | 1.8 ± 0.5 | 23.5 ± 12.2 | 54.2 ± 23.9 | 28.8 ± 15.1 | 89.1 ± 12.7 |
| 1. Dakar | 1.7 ± 0.9 | 20.9 ± 11.1 | 44.0 ± 17.9 | 25.2 ± 12.0 | 74.5 ± 12.6 |
| 1. Damascus | 2.9 ± 0.6 | 12.9 ± 3.9 | 24.2 ± 7.8 | 16.3 ± 5.3 | 55.4 ± 16.5 |
| 1. Djibouti | 2.4 ± 0.5 | 20.9 ± 6.7 | 44.3 ± 13.5 | 25.6 ± 7.8 | 72.4 ± 14.9 |
| 1. Doha | 1.8 ± 0.7 | 21.7 ± 8.5 | 47.8 ± 17.0 | 26.7 ± 10.7 | 86.2 ± 11.9 |
| 1. El-Aiun | 2.8 ± 0.7 | 5.5 ± 1.7 | 7.7 ± 2.8 | 5.7 ± 2.1 | 14.3 ± 7.0 |
| 1. Freetown | 2.0 ± 0.8 | 30.3 ± 17.3 | 63.3 ± 20.0 | 36.0 ± 17.3 | 94.3 ± 11.0 |
| **CITY** | **PERCENTAGE OF DAYS UNDER HEAT WAVE CONDITIONS** | | | | |
|  | **LATE 20^TH^ CENTURY**  **(1970-1999)** | **RCP4.5**  **(2020-2049)** | **RCP4.5**  **(2070-2099)** | **RCP8.5**  **(2020-2049)** | **RCP8.5**  **(2070-2099)** |
| 1. Giza | 2.7 ± 0.5 | 13.1 ± 6.3 | 23.5 ± 14.1 | 16.2 ± 7.9 | 50.2 ± 15.1 |
| 1. Istanbul | 1.3 ± 0.4 | 11.3 ± 8.2 | 15.8 ± 11.6 | 13.2 ± 9.5 | 38.5 ± 19.3 |
| 1. Jerusalem | 2.6 ± 0.5 | 8.8 ± 3.9 | 16.6 ± 9.5 | 11.1 ± 4.9 | 42.2 ± 20.5 |
| 1. Juba | 2.2 ± 0.6 | 26.7 ± 12.0 | 50.3 ± 18.9 | 32.6 ± 14.3 | 78.6 ± 11.9 |
| 1. Khartoum | 2.2 ± 0.6 | 22.2 ± 10.5 | 45.6 ± 15.2 | 27.9 ± 10.9 | 74.1 ± 12.8 |
| 1. Kuwait City | 1.2 ± 0.4 | 18.9 ± 7.5 | 38.2 ± 14.1 | 22.9 ± 9.4 | 69.7 ± 14.0 |
| 1. Lagos | 1.8 ± 0.6 | 35.6 ± 15.6 | 67.1 ± 21.2 | 41.0 ± 17.3 | 91.9 ± 9.7 |
| 1. Lome | 1.9 ± 0.6 | 33.0 ± 16.2 | 62.5 ± 20.5 | 36.8 ± 17.5 | 90.2 ± 8.4 |
| 1. Manama | 1.5 ± 0.5 | 17.9 ± 6.8 | 43.0 ± 13.6 | 23.9 ± 9.6 | 85.9 ± 9.5 |
| 1. Mogadishu | 2.5 ± 1.0 | 32.5 ± 10.6 | 69.3 ± 14.0 | 42.0 ± 12.1 | 96.0 ± 4.6 |
| 1. Monrovia | 1.5 ± 0.5 | 23.3 ± 9.9 | 50.3 ± 19.9 | 27.9 ± 11.3 | 85.4 ± 11.4 |
| 1. Muscat | 1.8 ± 0.4 | 18.5 ± 6.5 | 42.6 ± 14.2 | 25.1 ± 8.1 | 80.1 ± 10.9 |
| 1. N’Djamena | 2.4 ± 0.7 | 19.3 ± 6.4 | 37.2 ± 10.7 | 24.1 ± 8.1 | 62.7 ± 12.7 |
| **CITY** | **PERCENTAGE OF DAYS UNDER HEAT WAVE CONDITIONS** | | | | |
|  | **LATE 20^TH^ CENTURY**  **(1970-1999)** | **RCP4.5**  **(2020-2049)** | **RCP4.5**  **(2070-2099)** | **RCP8.5**  **(2020-2049)** | **RCP8.5**  **(2070-2099)** |
| 1. Niamey | 2.4 ± 0.8 | 21.6 ± 7.4 | 41.8 ± 12.7 | 25.5 ± 8.3 | 67.1 ± 13.3 |
| 1. Nouakchott | 1.6 ± 0.8 | 8.5 ± 2.7 | 16.2 ± 7.2 | 9.2 ± 2.6 | 32.3 ± 11.5 |
| 1. Ouagadougou | 2.4 ± 0.8 | 20.4 ± 6.8 | 40.7 ± 13.8 | 24.0 ± 8.8 | 66.8 ± 14.7 |
| 1. Porto-Novo | 1.8 ± 0.6 | 28.4 ± 12.6 | 56.9 ± 17.3 | 32.5 ± 14.3 | 86.7 ± 8.8 |
| 1. Rabat | 2.1 ± 0.7 | 7.0 ± 2.7 | 11.6 ± 4.8 | 7.6 ± 3.0 | 20.7 ± 9.1 |
| 1. Riyadh | 2.0 ± 0.6 | 37.4 ± 14.1 | 69.2 ± 20.0 | 44.7 ± 15.4 | 93.3 ± 9.9 |
| 1. Sanaa | 2.4 ± 0.7 | 25.7 ± 12.9 | 50.6 ± 20.3 | 30.6 ± 13.4 | 77.7 ± 17.5 |
| 1. Tehran | 2.1 ± 0.7 | 20.3 ± 9.4 | 43.8 ± 18.8 | 25.5 ± 12.1 | 77.1 ± 15.7 |
| 1. Tbilisi | 2.9 ± 0.8 | 15.7 ± 5.6 | 32.2 ± 12.7 | 19.3 ± 6.9 | 64.8 ± 17.1 |
| 1. Tripoli | 2.4 ± 0.6 | 5.3 ± 1.9 | 10.4 ± 5.3 | 6.8 ± 2.1 | 25.8 ± 15.3 |
| 1. Tunis | 2.1 ± 0.4 | 7.2 ± 5.1 | 15.4 ± 12.6 | 8.4 ± 5.9 | 37.6 ± 21.3 |
| 1. Yamoussoukro | 2.2 ± 0.8 | 17.4 ± 6.8 | 37.8 ± 12.7 | 20.7 ± 6.9 | 69.9 ± 12.0 |
| 1. Yaounde | 1.4 ± 0.4 | 18.5 ± 8.0 | 42.4 ± 17.3 | 22.6 ± 10.6 | 73.8 ± 11.5 |
| **CITY** | **PERCENTAGE OF DAYS UNDER HEAT WAVE CONDITIONS** | | | | |
|  | **LATE 20^TH^ CENTURY**  **(1970-1999)** | **RCP4.5**  **(2020-2049)** | **RCP4.5**  **(2070-2099)** | **RCP8.5**  **(2020-2049)** | **RCP8.5**  **(2070-2099)** |
| 1. Yerevan | 2.9 ± 0.5 | 16.0 ± 6.2 | 34.1 ± 13.0 | 20.2 ± 7.5 | 67.8 ± 17.3 |

**Table S4.** Percentage of days (value ± SD) under heat wave conditions per month and averaged over the periods 1970-1999, 2020-2049 and 2070-2099. RCP4.5 and RCP8.5 refer to the two representative concentration pathways used during the calculations. *All the results are statistically significant at more than 95%.*
