# Supplementary material for: Persistent heat waves projected for Middle East and North Africa by the end of the 21st century
Source: PLoS One. 2020 Nov 17;15(11):e0242477. doi: 10.1371/journal.pone.0242477 (PMC7671526; doi:10.1371/journal.pone.0242477)
Supplement: S5 Table — RCP4.5 and RCP8.5 refer to the two representative concentration pathways used during the calculations. All the results are statistically significant at more than 95%. (DOCX) [file pone.0242477.s005.docx]

| **CITY** | **HEAT WAVES INTENSITY** | | | | | | | | | | |
| --- | --- | --- | --- | --- | --- | --- | --- | --- | --- | --- | --- |
|  | **LATE 20^TH^ CENTURY**  **(1970-1999)** | | | **RCP4.5**  **(2020-2049)** | | **RCP4.5**  **(2070-2099)** | | **RCP8.5**  **(2020-2049)** | | **RCP8.5**  **(2070-2099)** | |
|  | **Mean** | | **Max** | **Mean** | **Max** | **Mean** | **Max** | **Mean** | **Max** | **Mean** | **Max** |
| 1. Abidjan | 0.9 ± 0.7 | | 3.3 ± 1.0 | 1.2 ± 0.9 | 4.6 ± 1.0 | 1.7 ± 1.1 | 5.9 ± 1.2 | 1.3 ± 0.9 | 5.0 ± 1.1 | 3.0 ± 1.4 | 7.8 ± 1.2 |
| 1. Abu Dhabi | 0.6 ± 0.4 | | 1.9 ± 0.5 | 1.0 ± 0.7 | 3.8 ± 0.4 | 1.5 ± 0.9 | 4.5 ± 0.5 | 1.1 ± 0.7 | 4.1 ±0.43 | 2.8 ± 1.4 | 7.2 ± 0.6 |
| 1. Abuja | 0.7 ± 0.5 | | 2.0 ± 0.4 | 1.0 ± 0.7 | 3.9 ± 0.5 | 1.4 ± 0.9 | 5.1 ± 0.7 | 1.1 ± 0.8 | 4.7 ± 1.2 | 2.6 ± 1.4 | 7.4 ± 1.0 |
| 1. Accra | 0.5 ± 0.4 | | 1.4 ± 0.5 | 0.7 ± 0.5 | 2.7 ± 0.7 | 1.1 ± 0.6 | 3.3 ± 0.5 | 0.8 ± 0.5 | 3.0 ± 0.8 | 2.3 ± 0.9 | 5.2 ± 0.8 |
| 1. Addis Ababa | 0.5 ± 0.4 | | 1.6 ± 0.4 | 1.0 ± 0.7 | 3.6 ± 0.5 | 1.6 ± 0.9 | 4.6 ± 0.6 | 1.2 ± 0.8 | 4.2 ± 0.9 | 3.1 ± 1.4 | 7.4 ± 1.0 |
| 1. Alexandria | 1.5 ± 1.2 | | 8.6 ± 2.4 | 1.6 ±1.4 | 9.8 ± 2.0 | 1.9 ± 1.7 | 12.0± 2.9 | 1.7 ± 1.6 | 10.9 ± 25 | 2.7 ± 1.9 | 14.0 ± 2.8 |
| 1. Algiers | 1.4 ± 0.9 | | 4.0 ± 1.1 | 1.7 ± 1.2 | 5.9 ± 1.1 | 1.9 ± 1.3 | 6.6 ± 1.3 | 1.8 ± 1.2 | 6.2 ± 1.4 | 2.7 ± 1.8 | 9.1 ± 1.4 |
| 1. Amman | 1.5 ± 1.0 | | 4.5 ± 0.7 | 2.0 ± 1.4 | 6.9 ± 1.4 | 2.1 ± 1.6 | 8.0 ± 1.3 | 2.0 ± 1.5 | 7.2 ± 1.4 | 2.7 ± 2.0 | 10.9 ± 1.6 |
| 1. Ankara | 1.5 ± 1.1 | | 4.9 ± 1.2 | 1.9 ± 1.4 | 6.6 ± 0.9 | 2.2 ± 1.6 | 8.3 ± 1.2 | 2.0 ± 1.5 | 7.3 ± 1.7 | 3.5 ± 2.2 | 11.8 ± 1.9 |
| 1. Ashgabat | 1.4 ± 1.0 | | 4.0 ± 1.2 | 1.7 ± 1.3 | 6.1 ± 1.2 | 2.1 ± 1.5 | 7.8 ± 1.4 | 1.9 ± 1.3 | 6.5 ± 0.9 | 3.1 ± 2.0 | 10.3 ± 1.4 |
| 1. Asmara | 0.7 ± 0.5 | | 2.3 ± 0.6 | 1.1 ± 0.8 | 4.1 ± 0.7 | 1.6 ± 1.0 | 5.5 ± 1.1 | 1.2 ± 0.8 | 4.8 ± 1.1 | 3.0 ± 1.5 | 8.3 ± 1.6 |
| **CITY** | **HEAT WAVES INTENSITY** | | | | | | | | | | |
|  | **LATE 20^TH^ CENTURY**  **(1970-1999)** | | | **RCP4.5**  **(2020-2049)** | | **RCP4.5**  **(2070-2099)** | | **RCP8.5**  **(2020-2049)** | | **RCP8.5**  **(2070-2099)** | |
|  | **Mean** | | **Max** | **Mean** | **Max** | **Mean** | **Max** | **Mean** | **Max** | **Mean** | **Max** |
| 1. Baghdad | 0.9 ± 0.6 | | 3.0 ± 1.0 | 1.5 ± 1.0 | 5.1 ± 0.6 | 1.9 ± 1.2 | 6.1 ± 1.0 | 1.6 ± 1.1 | 5.4 ± 0.9 | 3.2 ± 1.9 | 9.3 ± 0.9 |
| 1. Baku | 1.1 ± 0.8 | | 3.5 ± 0.9 | 1.5 ± 1.1 | 5.7 ± 0.9 | 1.8 ± 1.3 | 6.4 ± 0.8 | 1.6 ± 1.1 | 6.0 ± 1.1 | 2.6 ± 1.7 | 9.1 ± 1.5 |
| 1. Bamako | 0.7 ± 0.5 | | 2.3 ± 0.7 | 1.1 ± 0.7 | 3.8 ± 0.6 | 1.5 ± 0.9 | 5.0 ± 1.0 | 1.2 ± 0.8 | 4.5 ± 0.9 | 2.9 ± 1.4 | 7.5 ± 1.2 |
| 1. Bangui | 0.8 ± 0.6 | | 2.6 ± 0.4 | 1.1 ± 0.8 | 4.5 ± 0.8 | 1.6 ± 1.0 | 5.7 ± 0.8 | 1.3 ± 0.9 | 5.3 ± 0.9 | 2.8 ± 1.5 | 8.3 ± 1.2 |
| 1. Banjul | 0.7 ± 0.5 | | 2.1 ± 0.6 | 1.0 ± 0.7 | 3.6 ± 0.6 | 1.3 ± 0.9 | 4.6 ± 1.0 | 1.1 ± 0.8 | 4.3 ± 0.8 | 2.1 ± 1.3 | 6.5 ± 0.6 |
| 1. Beirut | 1.2 ± 0.9 | | 4.0 ± 0.8 | 1.5 ± 1.2 | 5.9 ± 1.3 | 1.7 ± 1.3 | 6.8 ± 1.0 | 1.6 ± 1.2 | 6.5 ± 1.5 | 2.1 ± 1.5 | 9.1 ± 1.4 |
| 1. Bissau | 0.8 ± 0.6 | | 2.5 ± 0.5 | 1.0 ± 0.7 | 4.0 ± 0.9 | 1.2 ± 0.9 | 4.8 ± 1.0 | 1.1 ± 0.8 | 4.2 ± 0.8 | 1.9 ± 1.2 | 6.8 ± 1.2 |
| 1. Cairo | 1.5 ± 1.2 | | 5.1 ± 1.3 | 1.6 ± 1.3 | 7.0 ± 1.0 | 1.8 ± 1.4 | 8.2 ± 1.1 | 1.7 ± 1.4 | 8.0 ± 1.7 | 2.6 ± 1.7 | 10.9 ± 1.3 |
| 1. Conakry | 0.7 ± 0.5 | | 2.4 ± 0.7 | 0.7 ± 0.5 | 3.5 ± 0.5 | 1.0 ± 0.7 | 4.6 ± 0.7 | 0.7 ± 0.5 | 3.8 ± 0.9 | 1.8 ± 0.8 | 6.1 ± 1.3 |
| 1. Dakar | 0.7 ± 0.5 | | 2.4 ± 1.1 | 0.8 ± 0.6 | 4.2 ± 0.6 | 1.1 ± 0.8 | 4.9 ± 0.8 | 0.9 ± 0.7 | 4.2 ± 0.7 | 1.8 ± 1.0 | 6.5 ± 0.8 |
| 1. Damascus | 1.4 ± 0.9 | | 3.8 ± 0.8 | 1.9 ± 1.3 | 6.5 ± 0.8 | 2.1 ± 1.5 | 7.1 ± 0.8 | 1.9 ± 1.3 | 6.6 ± 0.8 | 2.9 ± 2.0 | 10.4 ± 1.4 |
| **CITY** | **HEAT WAVES INTENSITY** | | | | | | | | | | |
|  | **LATE 20^TH^ CENTURY**  **(1970-1999)** | | | **RCP4.5**  **(2020-2049)** | | **RCP4.5**  **(2070-2099)** | | **RCP8.5**  **(2020-2049)** | | **RCP8.5**  **(2070-2099)** | |
|  | **Mean** | | **Max** | **Mean** | **Max** | **Mean** | **Max** | **Mean** | **Max** | **Mean** | **Max** |
| 1. Djibouti | 0.6 ± 0.5 | | 1.8 ± 0.6 | 1.0 ± 0.7 | 3.4 ± 0.7 | 1.4 ± 0.9 | 4.7 ± 0.8 | 1.1 ± 0.7 | 3.9 ± 0.6 | 2.5 ± 1.4 | 7.0 ± 0.9 |
| 1. Doha | 0.7 ± 0.5 | | 2.3 ± 0.6 | 1.1 ± 0.8 | 3.9 ± 0.4 | 1.4 ± 1.0 | 5.1 ± 0.6 | 1.2 ± 0.8 | 4.3 ± 0.5 | 2.6 ± 1.4 | 7.6 ± 0.9 |
| 1. El-Aiun | 2.2 ± 1.6 | | 6.5 ± 1.4 | 2.3 ± 1.7 | 7.8 ± 1.6 | 2.6 ± 1.8 | 8.1 ± 1.6 | 2.5 ± 1.8 | 8.1 ± 1.7 | 2.9 ± 2.2 | 10.7 ± 1.7 |
| 1. Freetown | 0.6 ± 0.5 | | 2.0 ± 0.6 | 0.7 ± 0.5 | 3.0 ± 0.7 | 1.0 ± 0.6 | 4.1 ± 1.0 | 0.8 ± 0.5 | 3.4 ± 1.0 | 2.0 ± 0.8 | 5.7 ± 1.4 |
| 1. Giza | 1.7 ± 1.3 | | 6.9 ± 2.0 | 1.9 ± 1.5 | 8.3 ±1.4 | 2.3 ± 1.7 | 9.7 ± 1.5 | 2.0 ± 1.6 | 9.2 ± 2.1 | 3.3 ± 2.1 | 12.5 ± 1.4 |
| 1. Istanbul | 1.6 ± 1.2 | | 5.2 ± 2.1 | 2.0 ± 1.6 | 8.4 ± 2.1 | 2.3 ± 1.7 | 9.1 ± 2.4 | 2.1 ± 1.6 | 8.8 ±2..5 | 3.1 ± 2.2 | 12.5 ± 2.7 |
| 1. Jerusalem | 1.7 ± 1.2 | | 5.3 ± 1.0 | 2.2 ± 1.6 | 7.6 ± 1.4 | 2.3 ± 1.7 | 8.7 ± 1.5 | 2.2 ± 1.7 | 8.2 ± 1.4 | 2.6 ± 2.0 | 11.4 ± 1.5 |
| 1. Juba | 0.5 ± 0.4 | | 1.5 ± 0.4 | 0.9 ± 0.6 | 3.3 ± 0.5 | 1.4 ± 0.9 | 4.4 ± 0.7 | 1.1 ± 0.7 | 3.8 ± 0.6 | 2.7 ± 1.3 | 6.8 ± 1.1 |
| 1. Khartoum | 0.8 ± 0.6 | | 2.5 ± 0.6 | 1.2 ± 0.8 | 4.1 ± 0.5 | 1.4 ± 0.9 | 4.4 ± 0.7 | 1.3 ± 0.9 | 4.2 ± 1.0 | 3.0 ± 1.6 | 8.5 ± 1.5 |
| 1. Kuwait City | 0.8 ± 0.6 | | 2.2 ± 0.6 | 1.2 ± 0.8 | 4.2 ± 0.6 | 1.7 ± 1.1 | 5.3 ± 0.4 | 1.3 ± 0.9 | 4.6 ± 0.7 | 3.0 ± 1.7 | 8.1 ± 0.8 |
| 1. Lagos | 0.7 ± 0.5 | | 2.6 ± 1.1 | 1.0 ± 0.7 | 3.8 ± 0.9 | 1.5 ± 0.8 | 4.6 ± 0.6 | 1.1 ± 0.7 | 4.0 ± 0.9 | 2.8 ± 1.0 | 6.6 ± 1.1 |
| **CITY** | **HEAT WAVES INTENSITY** | | | | | | | | | | |
|  | **LATE 20^TH^ CENTURY**  **(1970-1999)** | | | **RCP4.5**  **(2020-2049)** | | **RCP4.5**  **(2070-2099)** | | **RCP8.5**  **(2020-2049)** | | **RCP8.5**  **(2070-2099)** | |
|  | **Mean** | | **Max** | **Mean** | **Max** | **Mean** | **Max** | **Mean** | **Max** | **Mean** | **Max** |
| 1. Lome | 0.4 ± 0.3 | | 1.4 ± 0.5 | 0.7 ± 0.5 | 2.7 ± 0.7 | 1.1 ± 0.6 | 3.4 ± 0.5 | 0.8 ± 0.5 | 2.9 ± 0.6 | 2.2 ± 0.9 | 5.2 ± 0.8 |
| 1. Manama | 1.0 ± 0.8 | | 3.2 ± 1.0 | 1.3 ± 1.0 | 5.5 ± 0.9 | 1.4 ± 1.1 | 6.6 ± 1.2 | 1.3 ± 1.0 | 5.7 ± 1.1 | 2.6 ± 1.5 | 9.1 ± 1.4 |
| 1. Mogadishu | 0.5 ± 0.4 | | 1.7 ± 0.6 | 0.7 ± 0.5 | 2.7 ± 0.5 | 1.0 ± 0.6 | 3.5 ± 0.6 | 0.7 ± 0.5 | 2.9 ± 0.6 | 2.2 ± 0.9 | 5.4 ± 0.9 |
| 1. Monrovia | 0.5 ± 0.4 | | 1.8 ± 0.5 | 0.8 ± 0.5 | 2.9 ± 0.4 | 1.1 ± 0.7 | 3.6 ± 0.6 | 0.8 ± 0.6 | 3.2 ± 0.8 | 2.1 ± 1.0 | 5.4 ± 0.8 |
| 1. Muscat | 0.8 ± 0.6 | | 2.5 ± 0.6 | 1.1 ± 0.8 | 4.1 ± 0.8 | 1.4 ± 0.9 | 5.0 ± 0.6 | 1.1 ± 0.8 | 4.3 ± 0.8 | 2.4 ± 1.4 | 7.5 ± 0.5 |
| 1. N’Djamena | 0.8 ± 0.6 | | 2.5 ± 0.6 | 1.2 ± 0.9 | 4.6 ± 0.8 | 1.7 ± 1.1 | 5.6 ± 1.0 | 1.4 ± 0.9 | 5.2 ± 1.1 | 2.9 ± 1.6 | 8.1 ± 1.0 |
| 1. Niamey | 0.8 ± 0.6 | | 2.2 ± 0.4 | 1.1 ± 0.8 | 4.2 ± 0.9 | 1.6 ± 1.0 | 5.6 ± 0.8 | 1.3 ± 0.9 | 4.8 ± 0.6 | 2.9 ± 1.5 | 7.7 ± 0.9 |
| 1. Nouakchott | 0.8 ± 0.6 | | 2.4 ± 0.8 | 1.2 ± 0.9 | 4.3 ± 1.0 | 1.5 ± 1.0 | 4.9 ± 1.3 | 1.3 ± 0.9 | 4.6 ± 1.3 | 2.2 ± 1.4 | 7.0 ± 1.1 |
| 1. Ouagadougou | 0.7 ± 0.5 | | 2.3 ± 0.5 | 1.1 ± 0.8 | 4.3 ± 0.8 | 1.6 ± 1.0 | 5.3 ± 0.8 | 1.3 ± 0.9 | 4.7 ± 0.6 | 2.8 ± 1.5 | 7.8 ± 1.0 |
| 1. Porto-Novo | 0.5 ± 0.4 | | 1.7 ± 0.8 | 0.7 ± 0.5 | 2.7 ± 0.5 | 1.1 ± 0.6 | 3.6 ± 0.5 | 0.8 ± 0.6 | 3.1 ± 0.6 | 2.2 ± 0.9 | 5.5 ± 0.7 |
| 1. Rabat | 1.4 ± 1.0 | 4.0 ± 1.3 | | 1.9 ± 1.3 | 5.9 ± 1.4 | 2.3 ± 1.5 | 7.0 ± 1.2 | 2.0 ± 1.4 | 6.4 ± 1.7 | 3.0 ± 2.0 | 9.6 ± 2.2 |
| 1. Riyadh | 0.6 ± 0.4 | 1.9 ± 0.6 | | 1.1 ± 0.7 | 4.0 ± 0.5 | 1.7 ± 1.0 | 5.1 ± 0.4 | 1.3 ± 0.8 | 4.4 ± 0.4 | 3.7 ± 1.4 | 8.0 ± 0.9 |
| 1. Sanaa | 0.7 ± 0.5 | 2.3 ± 1.2 | | 1.1 ± 0.8 | 4.1 ± 1.0 | 1.7 ± 1.0 | 5.6 ± 1.0 | 1.3 ± 0.9 | 4.8 ± 1.1 | 3.3 ± 1.6 | 8.4 ± 1.1 |
| **CITY** | **HEAT WAVES INTENSITY** | | | | | | | | | | |
|  | **LATE 20^TH^ CENTURY**  **(1970-1999)** | | | **RCP4.5**  **(2020-2049)** | | **RCP4.5**  **(2070-2099)** | | **RCP8.5**  **(2020-2049)** | | **RCP8.5**  **(2070-2099)** | |
|  | **Mean** | **Max** | | **Mean** | **Max** | **Mean** | **Max** | **Mean** | **Max** | **Mean** | **Max** |
| 1. Tehran | 1.0 ± 0.7 | 2.8 ± 0.7 | | 1.5 ± 1.1 | 5.5 ± 0.7 | 2.1 ± 1.3 | 7.3 ± 1.0 | 1.7 ± 1.2 | 6.1 ± 0.8 | 3.6 ± 1.9 | 9.9 ± 1.2 |
| 1. Tbilisi | 1.2 ± 0.8 | 3.7 ± 1.1 | | 1.8 ± 1.3 | 6.4 ± 1.5 | 2.3 ± 1.5 | 8.0 ± 1.6 | 2.0 ± 1.4 | 7.6 ± 1.6 | 3.6 ± 2.2 | 11.3 ± 2.2 |
| 1. Tripoli | 1.7 ± 1.2 | 5.2 ± 1.3 | | 2.2 ± 1.5 | 6.8 ± 1.4 | 2.4 ± 1.7 | 7.9 ± 1.4 | 2.2 ± 1.5 | 7.1 ± 1.5 | 2.9 ± 2.1 | 10.6 ± 1.4 |
| 1. Tunis | 1.7 ± 1.3 | 5.1 ± 1.2 | | 2.1 ± 1.5 | 6.9 ± 0.9 | 2.3 ± 1.7 | 8.8 v 1.9 | 2.2 ± 1.7 | 8.0 ± 1.3 | 2.8 ± 2.0 | 10.7 ± 1.3 |
| 1. Yamoussoukro | 0.7 ± 0.5 | 2.3 ± 0.7 | | 1.0 ± 0.7 | 3.9 ± 0.6 | 1.3 ± 0.9 | 5.4 ± 1.5 | 1.0 ± 0.8 | 4.5 ± 1.5 | 2.4 ± 1.3 | 6.9 ± 1.2 |
| 1. Yaounde | 0.7 ± 0.5 | 2.0 ± 0.4 | | 0.9 ± 0.7 | 3.6 ± 0.5 | 1.3 ± 0.8 | 4.8 ± 0.7 | 1.0 ± 0.7 | 4.2 ± 0.8 | 2.4 ± 1.2 | 7.1 ± 1.0 |
| 1. Yerevan | 1.1 ± 0.8 | 3.7 ± 1.0 | | 1.8 ± 1.3 | 6.4 ± 1.3 | 2.3 ± 1.5 | 7.8 ± 1.4 | 2.0 ± 1.4 | 7.1 ± 0.9 | 3.7 ± 2.2 | 11.7 ± 1.7 |

**Table S5.** Mean and maximum intensity of days (value ± SD) under heat wave conditions and averaged over the periods 1970-1999, 2020-2049 and 2070-2099. RCP4.5 and RCP8.5 refer to the two representative concentration pathways used during the calculations. *All the results are statistically significant at more than 95%.*
