# Supplementary material for: Persistent heat waves projected for Middle East and North Africa by the end of the 21st century
Source: PLoS One. 2020 Nov 17;15(11):e0242477. doi: 10.1371/journal.pone.0242477 (PMC7671526; doi:10.1371/journal.pone.0242477)
Supplement: S6 Table — RCP4.5 and RCP8.5 refer to the two representative concentration pathways used during the calculations. All the results are statistically significant at more than 95%. (DOCX) [file pone.0242477.s006.docx]

| **CITY** | **CUMULATIVE INDEX** | | | |  |
| --- | --- | --- | --- | --- | --- |
|  | **RCP4.5**  **(2020-2049)** | **RCP4.5**  **(2070-2099)** | **RCP8.5**  **(2020-2049)** | **RCP8.5**  **(2070-2099)** | |
| 1. Abidjan | 0.3 ± 0.1 | 0.9 ± 0.2 | 0.4 ± 0.1 | 2.5 ± 0.2 | |
| 1. Abu Dhabi | 0.2 ± 0.1 | 0.7 ± 0.1 | 0.3 ± 0.1 | 2.3 ± 0.2 | |
| 1. Abuja | 0.2 ± 0.0 | 0.5 ± 0.1 | 0.3 ± 0.1 | 1.8 ± 0.2 | |
| 1. Accra | 0.2 ± 0.1 | 0.7 ± 0.1 | 0.3 ± 0.1 | 2.1 ± 0.1 | |
| 1. Addis Ababa | 0.3 ± 0.1 | 1.0 ± 0.2 | 0.5 ± 0.1 | 2.6 ± 0.2 | |
| 1. Alexandria | 0.2 ± 0.1 | 0.4 ± 0.2 | 0.3 ± 0.1 | 1.3 ± 0.3 | |
| 1. Algiers | 0.1 ± 0.1 | 0.4 ± 0.2 | 0.2 ± 0.1 | 1.3 ± 0.4 | |
| 1. Amman | 0.2 ± 0.1 | 0.4 ± 0.1 | 0.3 ± 0.1 | 1.3 ± 0.4 | |
| 1. Ankara | 0.3 ± 0.1 | 0.6 ± 0.2 | 0.3 ± 0.1 | 2.3 ± 0.3 | |
| 1. Ashgabat | 0.2 ± 0.1 | 0.6 ± 0.2 | 0.3 ± 0.1 | 1.8 ± 0.4 | |
| 1. Asmara | 0.3 ± 0.1 | 0.9 ± 0.2 | 0.4 ± 0.1 | 2.4 ± 0.2 | |
| 1. Baghdad | 0.3 ± 0.1 | 0.7 ± 0.2 | 0.4 ± 0.1 | 2.3 ± 0.3 | |
| 1. Baku | 0.2 ± 0.1 | 0.4 ± 0.2 | 0.2 ± 0.1 | 1.3 ± 0.3 | |
| **CITY** | **CUMULATIVE INDEX** | | | |  |
|  | **RCP4.5**  **(2020-2049)** | **RCP4.5**  **(2070-2099)** | **RCP8.5**  **(2020-2049)** | **RCP8.5**  **(2070-2099)** | |
| 1. Bamako | 0.3 ± 0.1 | 0.7 ± 0.1 | 0.3 ± 0.1 | 2.1 ± 0.2 | |
| 1. Bangui | 0.2 ± 0.1 | 0.6 ± 0.1 | 0.3 ± 0.1 | 1.8 ± 0.2 | |
| 1. Banjul | 0.2 ± 0.0 | 0.4 ± 0.1 | 0.2 ± 0.0 | 1.3 ± 0.2 | |
| 1. Beirut | 0.2 ± 0.1 | 0.4 ± 0.2 | 0.2 ± 0.1 | 1.2 ± 0.3 | |
| 1. Bissau | 0.1 ± 0.0 | 0.3 ± 0.1 | 0.1 ± 0.1 | 1.2 ± 0.2 | |
| 1. Cairo | 0.2 ± 0.1 | 0.5 ± 0.2 | 0.3 ± 0.1 | 1.6 ± 0.3 | |
| 1. Conakry | 0.2 ± 0.1 | 0.5 ± 0.2 | 0.2 ± 0.1 | 1.6 ± 0.1 | |
| 1. Dakar | 0.2 ± 0.1 | 0.5 ± 0.1 | 0.2 ± 0.1 | 1.3 ± 0.1 | |
| 1. Damascus | 0.2 ± 0.1 | 0.5 ± 0.1 | 0.3 ± 0.1 | 1.6 ± 0.3 | |
| 1. Djibouti | 0.2 ± 0.0 | 0.6 ± 0.1 | 0.3 ± 0.1 | 1.8 ± 0.2 | |
| 1. Doha | 0.2 ± 0.1 | 0.7 ± 0.2 | 0.3 ± 0.1 | 2.2 ± 0.2 | |
| 1. El-Aiun | 0.1 ± 0.0 | 0.2 ± 0.0 | 0.1 ± 0.0 | 0.4 ± 0.2 | |
| 1. Freetown | 0.2 ± 0.1 | 0.6 ± 0.1 | 0.3 ± 0.1 | 1.9 ± 0.1 | |
| **CITY** | **CUMULATIVE INDEX** | | | |  |
|  | **RCP4.5**  **(2020-2049)** | **RCP4.5**  **(2070-2099)** | **RCP8.5**  **(2020-2049)** | **RCP8.5**  **(2070-2099)** | |
| 1. Giza | 0.2 ± 0.1 | 0.5 ± 0.2 | 0.3 ± 0.1 | 1.6 ± 0.3 | |
| 1. Istanbul | 0.2 ± 0.1 | 0.4 ± 0.2 | 0.3 ± 0.1 | 1.2 ± 0.4 | |
| 1. Jerusalem | 0.2 ± 0.1 | 0.4 ± 0.2 | 0.2 ± 0.1 | 1.1 ± 0.4 | |
| 1. Juba | 0.2 ± 0.1 | 0.7 ± 0.2 | 0.4 ± 0.1 | 2.1 ± 0.2 | |
| 1. Khartoum | 0.3 ± 0.1 | 0.6 ± 0.1 | 0.4 ± 0.1 | 2.2 ± 0.2 | |
| 1. Kuwait City | 0.2 ± 0.1 | 0.6 ± 0.2 | 0.3 ± 0.1 | 2.1 ± 0.2 | |
| 1. Lagos | 0.4 ± 0.1 | 1.0 ± 0.2 | 0.4 ± 0.1 | 2.5 ± 0.1 | |
| 1. Lome | 0.2 ± 0.1 | 0.7 ± 0.1 | 0.3 ± 0.1 | 2.0 ± 0.1 | |
| 1. Manama | 0.2 ± 0.1 | 0.6 ± 0.1 | 0.3 ± 0.1 | 2.2 ± 0.1 | |
| 1. Mogadishu | 0.2 ± 0.1 | 0.7 ± 0.1 | 0.3 ± 0.1 | 2.1 ± 0.0 | |
| 1. Monrovia | 0.2 ± 0.0 | 0.6 ± 0.1 | 0.2 ± 0.1 | 1.8 ± 0.1 | |
| 1. Muscat | 0.2 ± 0.1 | 0.6 ± 0.1 | 0.3 ± 0.1 | 1.9 ± 0.2 | |
| 1. N’Djamena | 0.2 ± 0.1 | 0.6 ± 0.1 | 0.3 ± 0.1 | 1.8 ± 0.2 | |
| **CITY** | **CUMULATIVE INDEX** | | | |  |
|  | **RCP4.5**  **(2020-2049)** | **RCP4.5**  **(2070-2099)** | **RCP8.5**  **(2020-2049)** | **RCP8.5**  **(2070-2099)** | |
| 1. Niamey | 0.2 ± 0.1 | 0.7 ± 0.1 | 0.3 ± 0.1 | 1.9 ± 0.2 | |
| 1. Nouakchott | 0.1 ± 0.0 | 0.2 ± 0.1 | 0.1 ± 0.0 | 0.7 ± 0.2 | |
| 1. Ouagadougou | 0.2 ± 0.1 | 0.7 ± 0.1 | 0.3 ± 0.1 | 1.9 ± 0.2 | |
| 1. Porto-Novo | 0.2 ± 0.1 | 0.6 ± 0.1 | 0.3 ± 0.1 | 1.9 ± 0.1 | |
| 1. Rabat | 0.1 ± 0.0 | 0.3 ± 0.1 | 0.2 ± 0.0 | 0.6 ± 0.2 | |
| 1. Riyadh | 0.4 ± 0.1 | 1.2 ± 0.2 | 0.6 ± 0.1 | 3.5 ± 0.1 | |
| 1. Sanaa | 0.3 ± 0.1 | 0.9 ± 0.2 | 0.4 ± 0.1 | 2.6 ± 0.3 | |
| 1. Tehran | 0.3 ± 0.1 | 0.9 ± 0.2 | 0.4 ± 0.1 | 2.8 ± 0.3 | |
| 1. Tbilisi | 0.3 ± 0.1 | 0.7 ± 0.2 | 0.4 ± 0.1 | 2.3 ± 0.4 | |
| 1. Tripoli | 0.1 ± 0.0 | 0.2 ± 0.1 | 0.2 ± 0.0 | 0.7 ± 0.3 | |
| 1. Tunis | 0.2 ± 0.1 | 0.4 ± 0.2 | 0.2 ± 0.1 | 1.1 ± 0.4 | |
| 1. Yamoussoukro | 0.2 ± 0.0 | 0.5 ± 0.1 | 0.2 ± 0.1 | 1.7 ± 0.2 | |
| 1. Yaounde | 0.2 ± 0.1 | 0.6 ± 0.1 | 0.2 ± 0.1 | 1.8 ± 0.1 | |
| **CITY** | **CUMULATIVE INDEX** | | | |  |
|  | **RCP4.5**  **(2020-2049)** | **RCP4.5**  **(2070-2099)** | **RCP8.5**  **(2020-2049)** | **RCP8.5**  **(2070-2099)** | |
| 1. Yerevan | 0.3 ± 0.1 | 0.8 ± 0.2 | 0.4 ± 0.1 | 2.5 ± 0.4 | |

**Table S6.** Cumulative Index for the periods 2020-2049 and 2070-2099. RCP4.5 and RCP8.5 refer to the two representative concentration pathways used during the calculations. *All the results are statistically significant at more than 95%.*
