# Supplementary figures and images for: Persistent heat waves projected for Middle East and North Africa by the end of the 21st century
Source: PLoS One. 2020 Nov 17;15(11):e0242477. doi: 10.1371/journal.pone.0242477 (PMC7671526; doi:10.1371/journal.pone.0242477)

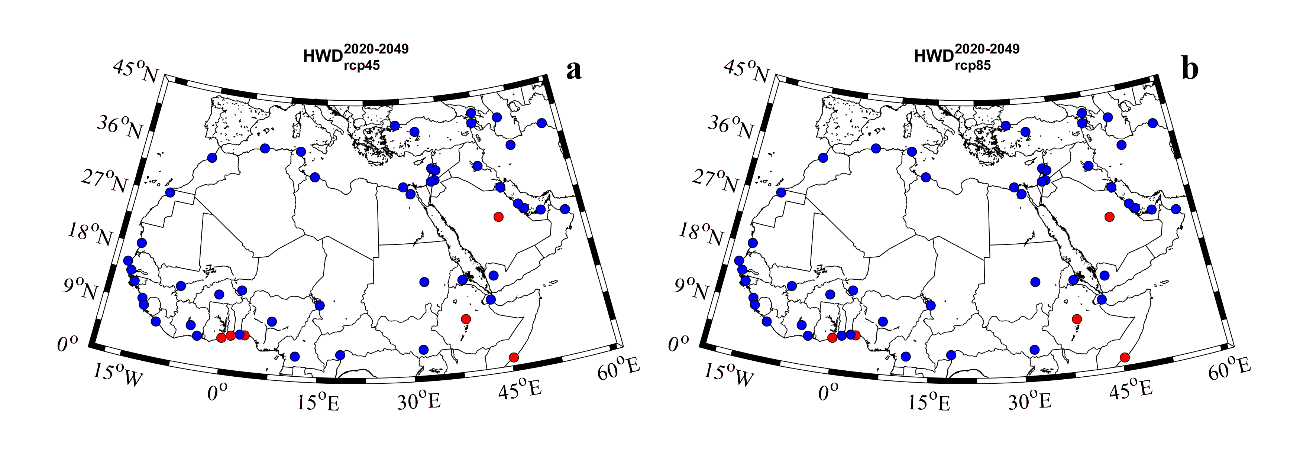

Supplement: S1 Fig — Cluster analysis of the percentages of heat wave days for near future (2020–2049) under the a) RCP45 scenario; b) RCP8.5 scenario. Blue color for low values and red color for high values. (TIF) [file pone.0242477.s007.tif]

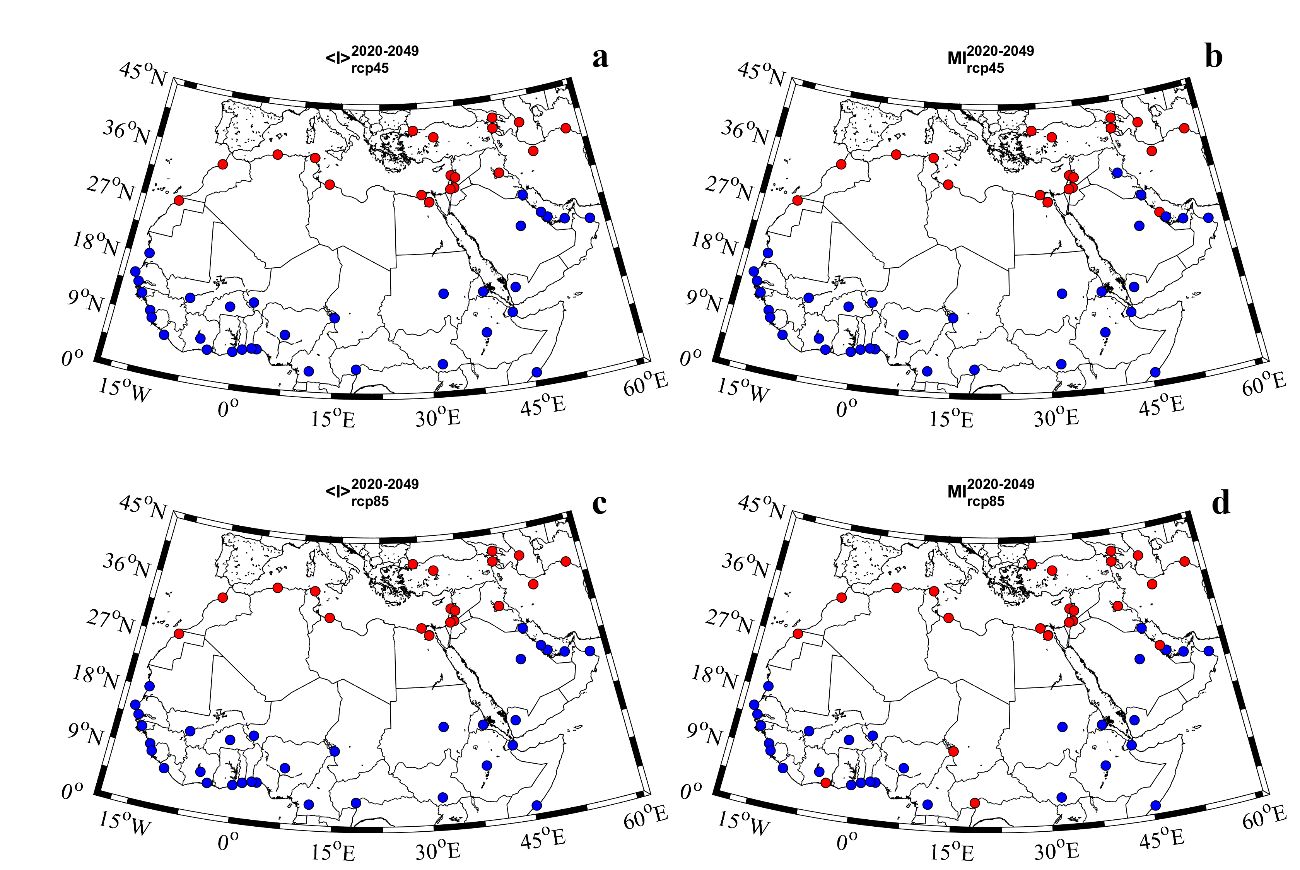

Supplement: S2 Fig — Cluster analysis of the mean (left panels) and maximum (right panels) heat wave intensity for near future (2020–2049) under the a, b) RCP45 scenario; c, d) RCP8.5 scenario. Blue color for low values and red color for high values. (TIF) [file pone.0242477.s008.tif]
