# Supplementary material for: Persistent heat waves projected for Middle East and North Africa by the end of the 21st century
Source: PLoS One. 2020 Nov 17;15(11):e0242477. doi: 10.1371/journal.pone.0242477 (PMC7671526; doi:10.1371/journal.pone.0242477)
Supplement: Attachment — Submitted filename: Review.pdf [file pone.0242477.s009.pdf]

This study has analyzed the duration and intensity of heat wave in the near-future and far-future for 53 cities in the Middle East and the North Africa (MENA) region for the 21st century. The output of 13 regional models were used to analyze the changes in extreme heat wave under two different scenarios (RCP4.5 and RCP8.5). Although I found the manuscript interesting, it needs revision. The introduction needs major revision.

- Merge two first paragraphs of the introduction into one.
- Introduction: Sentences are too long (e.g., 67-71) Make sentences shorted and more fluent.
- Line 29: Write numbers lower than 10 in letter, check it in all text
- Introduction needs more references for example line 33, 34, 44,
- Lina 35, Add the criterion to define the megacity so readers would have an idea of what you are talking about
- Line 39-43: Make sentences more fluent. For example: The required infrastructure services (e.g., clean water, electricity, transportation, etc) and the agglomeration of population, make cities highly vulnerable to climate change.
- Line 42: How the rapid increase of urbanization is increasing the number of people living in slums.
- Line 44: To say "On the other hand," you have to have "on one hand" phrase in previous sentences. Check it in the entire text.
- 54-59: The rise of temperature will lead to an increase in the frequency and persistent of heat waves in the future [References] with significant impact on human mortality [add reference here] and socio-economic conditions [references]
- Line 71, remove "in fact"
- Line 76 remove “as mentioned” and change the sentence to “The study area represent …”
- line 77 either add a reference or remove the sentence
- Line 78-80: modify the sentence as follow: In the past 20 years, MENA region showed the highest population gross in the world [reference]
- line 80 replace "on the other hand" with "besides"

- "Besides, the vast majority of MENA are characterized by frequent extreme temperature events (>35C) [add reference] and severe drought in summer [add reference], leading to large arid zones that may increase the risk of famine and famine-related mortality.
- line 89. change it to "Based on our knowledge now, "
- line 92-94. merge this sentence with the next sentence in line 95-96
- line 101. what near surface means?
- Data and method:
- How many grid-cell did you analyzed for each city?
- Did you consider the entire area of the city or just one point (center point of the city)?
- line 128, remove the numbering.
- line 139 after 2-6 days add references s\like:
  - Mazdiyasni, O., Aghakouchak, A., 2015. Substantial increase in concurrent droughts and heatwaves in the United States. Proc. Natl. Acad. Sci. 112, 11484–11489. https://doi.org/10.1073/pnas.1422945112.
  - Tavakol, Ameneh, Vahid Rahmani, and John Harrington Jr. "Evaluation of hot temperature extremes and heat waves in the Mississippi River Basin." Atmospheric Research 239 (2020): 104907. https://doi.org/10.1016/j.atmosres.2020.104907
  - Nissan, H., Burkart, K., Coughlan de Perez, E., Van Aalst, M., Mason, S., 2017. Defining and predicting Heat Waves in Bangladesh. J. Appl. Meteorol. Climatol. 56, 2653–2670. https://doi.org/10.1175/JAMC-D-17-0035.1.
- line 165: explain the parameters
- Add a figure showing what months you have considered for analysis in each city?
- What the distribution of months for different location means? Discuss it.

- Except for the statistics of changes in the frequency and intensity of events, you have to specify which regions will experience extreme events in the future. Summarize the spatial occurrence of most significant changes.
- I suggest you use clustering methods (e.g., average linkage, k-mean, etc.) to cluster stations based on the frequency and intensity of events
- Is there a statistically significant difference between the changes in the frequency and intensity of events in near and far future?
- Line 342: higher than what?
- How the distribution of different months for each location influenced the results.
- Figures:
- Add unit to the figures' legends
- Add scale to figures
- What HWD and IM means in table 1?
